# Vertical transmission of Zika virus in *Culex quinquefasciatus* Say and *Aedes aegypti* (L.) mosquitoes

**DOI:** 10.1038/s41598-019-41727-8

**Published:** 2019-03-27

**Authors:** Atchara Phumee, Jakkrawarn Chompoosri, Proawpilart Intayot, Rungfar Boonserm, Siwaporn Boonyasuppayakorn, Rome Buathong, Usavadee Thavara, Apiwat Tawatsin, Yutthana Joyjinda, Supaporn Wacharapluesadee, Padet Siriyasatien

**Affiliations:** 1 Thai Red Cross Emerging Infectious Health Science Centre, Neuroscience Center for Research and Development & WHO-CC for Research and Training on Viral Zoonoses King Chulalongkorn Memorial Hospital, Faculty of Medicine, Chulalongkorn University, Bangkok, 10330 Thailand; 2 Vector Biology and Vector Borne Disease Research Unit, Department of Parasitology, Faculty of Medicine, Chulalongkorn University, Bangkok, 10330 Thailand; 3 National Institute of Health, Department of Medical Sciences, Nonthaburi, 11000 Thailand; 4 Medical Science Program, Faculty of Medicine, Chulalongkorn University, Bangkok, 10330 Thailand; 5 Applied Medical Virology Research Unit, Department of Microbiology, Faculty of Medicine, Chulalongkorn University, Bangkok, 10330 Thailand; 6 Department of Disease Control, Bureau of Epidemiology, Ministry of Public Health, Nonthaburi, 11000 Thailand

## Abstract

Several mosquito species have been described as vectors for the Zika virus (ZIKV), such as those in the *Aedes*, *Anopheles*, *Mansonia* and *Culex* genera. Our previous survey studies were found the ZIKV RNA positive in both male, female and larvae of *Culex quinquefasciatus* Say and *Aedes aegypti* (L.) mosquitoes collected from active ZIKV infected patients’ homes in Thailand. Therefore, the aims of this study were to investigate whether ZIKV could be vertically transmitted in *Cx*. *quinquefasciatus*, *Ae*. *aegypti* and *Ae*. *albopictus*. Laboratory and field colonies of these mosquito species were maintained and artificially fed with ZIKV in human blood. Fully engorged mosquitoes (F_0_) were selected and reared for the vertical transmission study. The subsequent mosquito generations were fed with human blood without the virus. ZIKV in the mosquitoes was detected by hemi-nested RT-PCR and sequencing. C6/36 cells were used to isolate ZIKV from samples that tested positive by hemi-nested RT-PCR. Moreover, ZIKV was identified by immunocytochemical staining 7 days after infection in several organs of infected F_0_ females, including the salivary glands, midguts, yoke granules and facet cells of the eye. The localization of the ZIKV antigen was identified by the presence of the specific antibody in the salivary glands, midguts, yoke granules and facet cells. ZIKV was detected in female and male *Cx*. *quinquefasciatus* until the F_6_ and F_2_ generations, respectively. The isolated virus showed cytopathic effects in C6/36 cells by 5 days postinfection. The results suggested that the vertical transmission of ZIKV occurs in *Cx*. *quinquefasciatus* in the laboratory. However, we were able to detect the presence of ZIKV in *Ae*. *aegypti* in only the F_1_ generation in both male and female mosquitoes, and *Ae*. *albopictus* mosquitoes were not able to vertically transmit the virus at all. Data obtained from this study could be valuable for developing a better understanding of the role of *Cx*. *quinquefasciatus* as a potential vector for ZIKV transmission in Thailand and may be useful in creating more effective mosquito vector control strategies in the future.

## Introduction

Zika virus (ZIKV) is an arbovirus belonging to the Flaviviridae family and the *Flavivirus* genus, which includes African and Asian lineages^1,2^. ZIKV infection remains a serious public health threat, especially to pregnant women because of its close association with microcephaly and other severe neurological complications in the developing fetus^3^. In addition, ZIKV is also associated with Guillain-Barré syndrome (GBS)^4^. ZIKV in Uganda was first isolated from a febrile sentinel rhesus monkey in 1947 and from pooled specimens of *Aedes africanus* mosquitoes in 1948^5^. ZIKV is primarily transmitted by *Aedes* mosquitoes^4^. Previous studies reported that *Aedes* mosquitoes such as *Ae*. *africanus*, *Ae*. *aegypti*, *Ae*. *apicocoargenteus*, *Ae*. *furcifer*, *Ae*. *vittatus* and *Ae*. *luteocephalus* are the principal vectors of ZIKV^5–9^. Diallo *et al*. (2014) identified several mosquito species as probable vectors of ZIKV in Southeastern Senegal, such as *Ae*. *furcifer*, *Ae*. *luteocephalus*, *Ae*. *africanus*, *Ae*. *vittatus*, *Ae*. *taylori*, *Ae*. *dalzieli*, *Ae*. *hirsutus*, *Ae*. *metallicus*, *Ae*. *aegypti*, *Ae*. *unilineatus*, *Mansonia uniformis*, *Culex perfuscus* and *Anopheles coustani*, using virus isolation and reverse transcription-polymerase chain reaction (RT-PCR) techniques^10^. In Southeast Asia, ZIKV was isolated from wild-caught *Ae*. *aegypti* in Malaysia^11^; moreover, *Ae*. *aegypti*^12^ and *Ae*. *albopictus*^13^ were reported as potential vectors of ZIKV transmission in Singapore. However, knowledge of the vectors of ZIKV transmission in Thailand is limited. In Thailand a total of 686 of confirmed Zika case had been reported between January and November 2016^14^, by the Ministry of Public Health (MoPH), especially in Phitsanulok and Chanthaburi provinces where *Cx*. *quinquefasciatus* were found naturally ZIKV infections. In addition, the MoPH also reported the first two indigenous cases of ZIKV-related microcephaly in Thailand^15^. In 2018, the Bureau of Epidemiology (BoE) of the MoPH revealed that 306 Zika case were reported from 22 provinces in January-August 2018^16^. Our previous studies, ZIKV RNA have been found in 1.85% female, 1.66% male, and 0.29% larva of *Cx*. *quinquefasciatus* mosquitoes collected from active ZIKV infected patients’ homes are infected with ZIKV in Thailand^17^. Therefore, in this study, we determined the potential for the Thai *Cx*. *quinquefasciatus* mosquito to vertically transmit ZIKV. The hemi-nested RT-PCR (hnRT-PCR) developed for this study is able to effectively detect ZIKV in mosquitoes. Information obtained from this study provides fundamental data regarding whether *Ae*. *aegypti*, *Ae*. *albopictus* and *Cx*. *quinquefasciatus* are capable of vertically transmitting ZIKV in the laboratory. There are no vaccines or specific therapies against ZIKV. Data regarding ZIKV infection in these mosquitoes will be valuable in developing effective control strategies for ZIKV infection in Thailand.

## Results

### Vertical transmission of ZIKV in *Ae. aegypti, Ae. albopictus* and *Cx*. *quinquefasciatus*

The mosquitoes were maintained and artificially fed with 1.7 × 10^5^ florescent focus units (FFU)/ml of ZIKV in human blood. The experiments were performed in triplicate. Progeny of *Ae*. *aegypti*, *Ae*. *albopictus* and *Cx*. *quinquefasciatus* exposed to ZIKV were reared to subsequent generations. ZIKV RNA was detected in each pooled generation of mosquitoes.

In field strains of *Ae*. *aegypti*, a total of 120 pool consisting of 2,400 F_1_ female adults and 30 pool consisting of 900 F_1_ male adults were tested in triplicate. We found that the male and female mosquitoes exposed to ZIKV can maintain the virus for only one generation and were showed infected at rates of 3.3% and 3.3%, respectively. In the laboratory strain, two pools of female F1 progeny were positive with infection rate of 1.7%, and ZIKV was not detected in the F1 generation of the male mosquito (Table 1). In both the laboratory and field strains of *Ae*. *albopictus*, the results showed that ZIKV was not detected in the offspring.

**Table 1:** Percent infected via vertical transmission of ZIKV in each generation in both the field and laboratory strains of *Ae*. *aegypti* mosquitoes.

Strain	Sex (n) per replicate	Generation:F [Mean ± SD (% ZIKV infection)]	
		F1	F2
Laboratory	Female (40)	0.7 ± 1.2 (1.7)	0.0 ± 0.0 (0)
	Male (10)	0.0 ± 0.0 (0)	0.0 ± 0.0 (0)
Field	Female (40)	1.3 ± 0.6 (3.3)	0.0 ± 0.0 (0)
	Male (10)	0.3 ± 0.6 (3.3)	0.0 ± 0.0 (0)

In *Cx*. *quinquefasciatus*, ZIKV RNA could be detected until the F_6_ generation in females and the F_2_ generation in males. The F_1_ generation had the highest level of ZIKV infection (29.2%), which decreased to 24.2%, 8.3%, 7.5%, 5.8%, 0.83% and 0% in the F_2_ to F_7_ generations of female mosquitoes, respectively. However, in male mosquitoes, the transmission of ZIKV was only found by using hnRT-PCR until the F_2_ generation, decreasing from 20% in F_1_ to 16.7% in F_2_ and then undetectable levels in F_3_ (Table 2). The nucleotide sequences of all positive samples showed 99–100% similarity to the Zika virus/*H*. *sapiens*-tc/THA/2014/SV0127-14 (accession number KU681081) that was used to infect the F_0_ generation; this strain belongs to the Asian lineage of the ZIKV. The entire sequences were assigned GenBank numbers (GenBank: MK028538-MK028557).

**Table 2:** Percent infected via vertical transmission of ZIKV in each generation in laboratory strain of *Cx*. *quinquefasciatus* mosquitoes.

Sex (n) per replicate	Generation:F [Mean ± SD (% ZIKV infection)]						
	F_1_	F_2_	F_3_	F_4_	F_5_	F_6_	F_7_
Female (40)	12 ± 7.0 (29.2)	9.7 ± 6.8 (24.2)	3.3 ± 3.1 (8.3)	3.0 ± 2.0 (7.5)	2.3 ± 2.1 (5.8)	0.3 ± 0.6 (0.83)	0.0 ± 0.0 (0)
Male (10)	2 ± 2 (20.0)	1.7 ± 2.9 (16.7)	0.0 ± 0.0 (0)	0.0 ± 0.0 (0)	0.0 ± 0.0 (0)	0.0 ± 0.0 (0)	0.0 ± 0.0 (0)

### Isolation of positive ZIKV from each generation of mosquitoes in C6/36 cells

**Figure 1:**
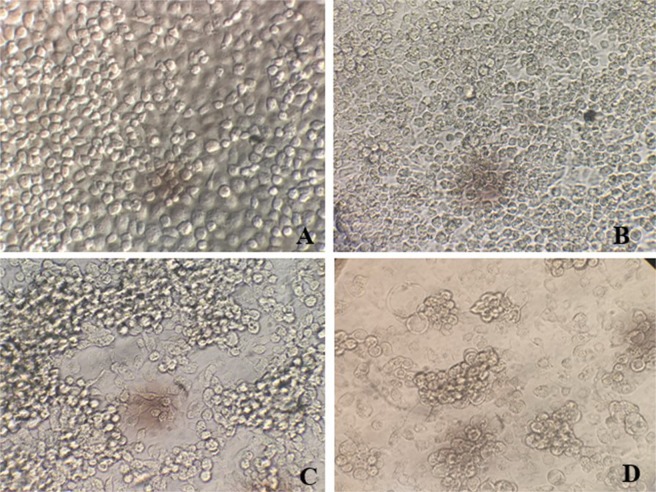
Cytopathic effects in C6/36 cells infected with ZIKV and not infected with ZIKV (**A**) and in C6/36 cells at postinfection day 3 (**B**), day 5 (**C**) and day 7 (**D**) under an inverted microscope (400x magnification).

Samples that were positive for ZIKV RNA in each generation of mosquitoes were used to isolate the virus by inoculating C6/36 cells, and the morphological changes of the infected cells were compared with the morphology of uninfected cells (Fig. 1A). The ZIKV infection in C6/36 was monitored microscopically for cytopathic effects (CPEs) at days 3 (Fig. 1B), 5 (Fig. 1C) and 7 (Fig. 1D) after inoculation. CPEs were observed on days 5–7 post-ZIKV inoculation, and ZIKV RNA was detected by hnRT-PCR. The characteristic CPEs of ZIKV infection are a loss of the normal cell shape, cell rounding, multinucleated giant cells, nuclear vacuolization, and degeneration of the cells. The results of this study suggest that ZIKV from mosquitoes can replicate in C6/36 cells. *Cx*. *quinquefasciatus* is clearly suspected of being able to vertically transmit ZIKV.

### Immunocytochemistry (ICC) staining of ZIKV in mosquitoes

**Figure 2:**
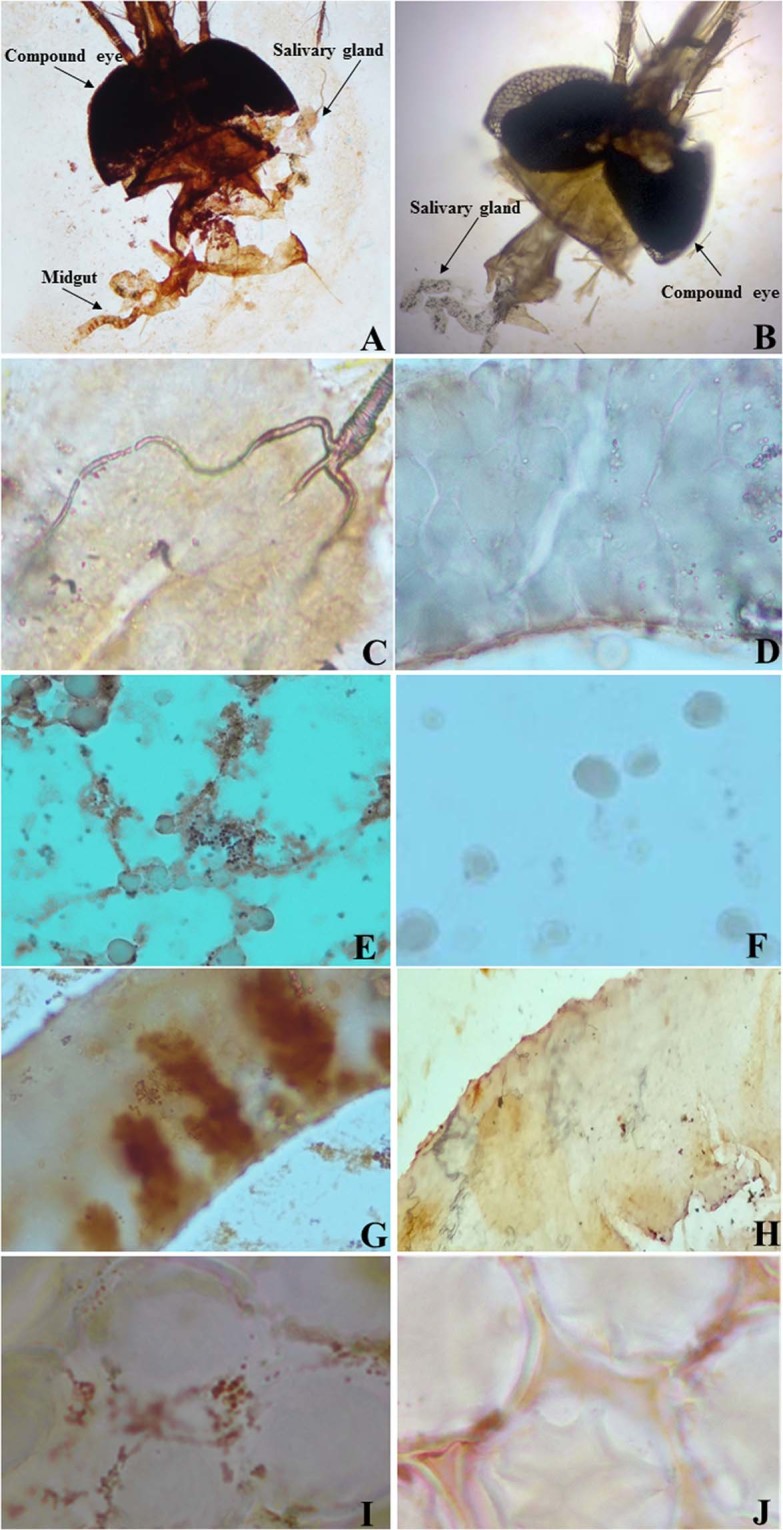
The ZIKV-infected F_0_ generation of *Cx*. *quinquefasciatus* mosquitoes at 7 days postinfection. The mosquitoes were infected via artificial blood feeding. Positive test results for the presence of the ZIKV antigen by using ICC staining of the positive head (**A**), negative head (**B**), positive salivary glands (**C**), negative salivary glands (**D**), positive midguts (**E**), negative midguts (**F**), positive yoke granules (**G**), negative yoke granules (**H**), positive facet cells (**I**) and negative facet cells (**J**).

The F_0_ generation of female *Cx*. *quinquefasciatus* mosquitoes that were infected with ZIKV were dissected 7 days postinfection to obtain their salivary glands, embryos, midguts, and heads. ZIKV antigens were detected in the salivary glands, midguts, yoke granules and facet cells of the eyes by ICC assay (Fig. 2). The dissection of the heads of the mosquitoes allowed investigation of the salivary glands, midguts and compound eyes. Positive staining for ZIKV is shown as distinct brown staining caused by the oxidation of 3,3′-diaminobenzidine (DAB) by horseradish peroxidase (HRP) within the organs of the mosquitoes (Fig. 2A). In the salivary glands, which are essential for transmission, ZIKV antigens were detected in the three lobes, especially in the distal lateral lobes, which play a major role in the blood feeding process (Fig. 2C). ZIKV can replicate in the midguts (Fig. 2E), yoke granules (Fig. 2G) and facet cells (Fig. 2I), all of which displayed localization of the ZIKV antigen-specific ZIKV-NS1 protein antibody, seen as brownish-red staining (Fig. 2A,C,E,G,I) compared with uninfected mosquitoes (Fig. 2B,D,F,H,J).

### ZIKV infection, dissemination and transmission rates in the F_0_ generations of *Ae*. *aegypti*, *Ae*. *albopictus*, and *Cx*. *quinquefasciatus* mosquitoes

The ICC results in F_0_ mosquitoes were used to calculate the infection, dissemination and transmission rates in the F_0_ generations of *Ae*. *aegypti*, *Ae*. *albopictus*, and *Cx*. *quinquefasciatus* mosquitoes. Mosquitoes that died before 7 days postinfection were excluded from the study. The *Ae*. *aegypti* laboratory strain and field strain had infection, dissemination and transmission rates of 88.2% and 90.5%, 71.1% and 67.4% and 60.8% and 60.0%, respectively. The *Ae*. *albopictus* laboratory strain and field strain had infection, dissemination and transmission rates of 53.9% and 51.0%, 41.8% and 42.0% and 21.8% and 22.0%, respectively. The *Cx*. *quinquefasciatus* laboratory strain had infection, dissemination and transmission rates of 87.5%, 72.4% and 63.3%, respectively (Table 3).

**Table 3:** Rates of ZIKV infection, dissemination and transmission in the F_0_ generation of *Ae*. *aegypti*, *Ae*. *albopictus*, and *Cx*. *quinquefasciatus* mosquitoes collected in Thailand.

Strains	Mosquitoes tested	7 dpi		
		Infection rate	Dissemination rate	transmission rates
Laboratory	*Cx*. *quinquefasciatus*	98:112 (87.5%)	71:98 (72.4%)	62:98 (63.3%)
	*Ae*. *aegypti*	97:110 (88.2%)	69:97 (71.1%)	59:97 (60.8%)
	*Ae*. *albopictus*	55:102 (53.9%)	23:55 (41.8%)	12:55 (21.8%)
Field	*Ae*. *aegypti*	95:105 (90.5%)	64:95 (67.4%)	57:95 (60.0%)
	*Ae*. *albopictus*	50:98 (51.0%)	21:50 (42.0%)	11:50 (22.0%)

### Calculation of the filial infection rate (FIR) of ZIKV in progeny

The FIR of ZIKV in the progeny was tested using pooled populations of mosquitoes; these pools consisted of 2,400 and 900 filial female and male adult mosquitoes, respectively. To calculate the FIR, the total number of pools was divided by the number of positive pools. Each positive pool indicated that one or more of the filial progeny in the pool were infected with ZIKV. In this study, female *Cx*. *quinquefasciatus* mosquitoes had an FIR for ZIKV of 1:66 for F1, which decreased to 1:2,400 in F6. Male *Cx*. *quinquefasciatus* mosquitoes had an FIR for ZIKV of 1:150 for F1, which decreased to 1:180 for F2. In the *Ae*. *aegypti* laboratory strain, the female mosquitoes had an FIR for F1 of 1:1,200, while the *Ae*. *aegypti* field strain female and male mosquitoes had FIRs for F1 of 1:600 and 1:900, respectively. The FIR results for ZIKV infection for the progeny are shown in Table 4.

**Table 4:** Filial infection rate (FIR) of ZIKV in the progeny of *Ae*. *aegypti* and *Cx*. *quinquefasciatus* mosquitoes.

Strains	Mosquitoes tested	Sex	Filial infection rate (s)					
			F1	F2	F3	F4	F5	F6
Laboratory	*Cx*. *quinquefasciatus*	Female	1:66	1:83	1:240	1:267	1:345	1:2,400
		Male	1:150	1:180	—	—	—	—
	*Ae*. *aegypti*	Female	1:1,200	—	—	—	—	—
		Male	—	—	—	—	—	—
Field	*Ae*. *aegypti*	Female	1:600	—	—	—	—	—
		Male	1:900	—	—	—	—	—

## Discussion

Our results demonstrated that vertical transmission of ZIKV occurs in the *Cx*. *quinquefasciatus* mosquito. The study was based on a molecular technique (hnRT-PCR) for ZIKV RNA detection, the isolation of viruses in cell culture and an ICC assay for the localization of a ZIKV-specific antigen. *Culex* mosquito species have been found to be able to vertically transmit West Nile (WNV)^18^ and Japanese encephalitis (JE)^19^ viruses. The role of the vertical transmission of ZIKV is still under investigation. Several reports have suggested that ZIKV could be transovarially transmitted to progeny in both laboratory and field experiments. Guo *et al*. (2016) revealed that *Cx*. *pipiens quinquefasciatus* clearly demonstrates the potential to be a vector for ZIKV in Southern China^20^. The *Cx*. *quinquefasciatus* laboratory colonies had detectable ZIKV in the midgut and salivary glands after artificial blood feeding with ZIKV; moreover, field-caught *Cx*. *quinquefasciatus* tested positive for ZIKV by RT-qPCR in Brazil^21^. In addition, *Cx*. *quinquefasciatus* had detectable ZIKV in the salivary glands at 7 and 15 days postfeeding in Northeast Brazil^22^.

In contrast, several reports have shown evidence of a lack of competence of *Culex* species for ZIKV^23,24^. For example, ZIKV isolated from *Cx*. *pipiens* and *Cx*. *quinquefasciatus* mosquitoes from the United States were unable to replicate, as determined by a plaque assay^25,26^. Similar reports showed that *C*. *pipiens* and other *Culex* species from Brazil^27^, Germany^28^, Tunisia^29^, Italy^30^ and Australia^31^ had no detectable ZIKV transmission. Factors that affect the competence of mosquito vectors include the following. (1) Differences in geographic regions mean that mosquito colonies from various areas have unique genetic backgrounds; therefore, mosquitoes collected from different areas may have varying degrees of viral competence^32–34^. This genetic variation may affect the morphology of mosquito organs and processes that are involved in virus replication and dissemination, such as mosquito immune responses, small RNA-based interferon (RNAi) pathways^35–37^ and the midgut and salivary gland barriers^29,38^. (2) Mosquitoes collected from different geographic regions also have different microbiomes and microviromes^39^. Microbiomes have been shown to interfere with viral replication in mosquito vectors; therefore, different microbiomes would affect the competency of the mosquitoes for ZIKV^40–42^. With regard to intracellular bacteria in mosquitoes, *Wolbachia* is another factor that affects viral replication in mosquitoes. A novel strategy to interfere with arbovirus transmission in mosquitoes using *Wolbachia pipientis* (wPip) has been proposed^43,44^. However, a study by Lourenço-de-Oliveira, *et al*. (2018) showed that no ZIKV was found in *Cx*. *quinquefasciatus* lines whether or not they contained *Wolbachia*^38^. The effects of the microbiome and *Wolbachia* in *Cx*. *quinquefasciatus* lines in Thailand could be investigated in the future. (3) The differences in the ZIKV strains used for the experiments, including the genotype, titer, and number of passages, could affect the ability of the ZIKV to enter mosquito organs, resulting in differences in replication and dissemination in the mosquitoes^32–44^. (4) The techniques used in the experiments, such as the mode of ZIKV infection of the mosquitoes (oral, intrathoracic) and the ZIKV detection method (plague assay, molecular techniques, immunological techniques), which have different sensitivities and specificities^32–44^, might also result in different findings.

This study is the first report of vertically transmitted ZIKV in *Cx*. *quinquefasciatus* and *Ae*. *aegypti* mosquitoes in Thailand. We also successfully isolated infectious ZIKV from a C6/36 cell line that had been infected with ZIKV from *Cx*. *quinquefasciatus* and and *Ae*. *aegypti*. *Cx*. *quinquefasciatus* mosquitoes are widely distributed in tropical and subtropical areas^45^. In Thailand, there have been reports of JE virus isolation from *Cx*. *quinquefasciatus* using C6/36 cells^46^. However, ZIKV in *Cx*. *quinquefasciatus* has never been studied in Thailand. In this study, we investigated the localization of ZIKV antigens in the organs of mosquitoes by ICC. The salivary gland, midguts, yoke granules and facet cells showed a reaction between the ZIKV antigen and its specific antibody. The salivary glands of mosquitoes play an important role in the transmission of pathogens and an essential role in the transmission cycle^47^. This study demonstrated that ZIKV also infects the salivary glands and midguts; therefore, we hypothesized that ZIKV was replicating in the salivary glands and midguts at 7 days postinfection. Several reports have shown the presence of ZIKV in the salivary glands and midguts of female mosquitoes such as *Ae*. *aegypti*^48^, *Ae*. *albopictus*^49^, *Cx*. *quinquefasciatus*^21^, *Cx*. *coronator*, and *Cx*. *tarsalis*^50^. The ICC assay was first performed on the facet cells and yoke granules of mosquitoes orally infected with ZIKV. The facet cells of the mosquito eyes showed brownish-red staining in the ICC assay. The ZIKV infection, dissemination and transmission rates in the F_0_ generations of *Ae*. *aegypti*, *Ae*. *albopictus*, and *Cx*. *quinquefasciatus* mosquitoes were calculated as shown in Table 3. Based on these results, there were no differences in the infection, dissemination or transmission rates in the F_0_ generations of the laboratory and field strains in both *Ae*. *aegypti* and *Ae*. *albopictus*. However, *Ae*. *aegypti* has higher infection, dissemination and transmission rates than *Ae*. *albopictus*, which is a similar finding to that in a recent report by Liu *et al*. (2017). High infection, dissemination and transmission rates in *Ae*. *aegypti* were also reported by Liu *et al*. (2017) and Main *et al*.^24,51^. In this study, we found lower infection, dissemination and transmission rates in *Ae*. *albopictus* than in *Ae*. *aegypti*, which is similar to the results of the study by Liu *et al*. (2017). Interestingly, the infection, dissemination and transmission rates of ZIKV in *Cx*. *quinquefasciatus* mosquitoes were higher in this study than in previous reports^24,51^. As discussed previously, several factors could affect the competency of mosquitoes for ZIKV, and future studies should be conducted to investigate the factors influencing these results. However, in this study we determined the transmission rates by examining virus infection in salivary glands, not in the expectorated saliva; therefore, the transmission rates in this study may over estimated. Further investigation for transmission rate in expectorated saliva should be perform for obtaining more accurate data.

The FIRs for ZIKV in the F1 generation of *Ae*. *aegypti* in both the laboratory and field strains in this study were lower than those in the previous reports by Thangamani *et al*.^52^ and Ciota *et al*.^53^, which had FIRs of 1:290 and 1:84, respectively. However, for *Cx*. *quinquefasciatus* mosquitoes, our results showed that the FIR for ZIKV in the F1 generation was high (1:66) and that it decreased to 1:2,400 in the F6 generation. The high FIR for ZIKV is related to the vertical transmission phenomenon.

The results of this study provide more information about the transmission dynamics of ZIKV in mosquitoes and could be used to explain the natural pathogenesis of ZIKV infection in wild mosquitoes. The presence of ZIKV antigens in the yoke granules may be associated with the vertical transmission of ZIKV, as ZIKV may infect the germinal tissues of the female mosquito and may occur in the fully formed egg during oviposition^54,55^. However, the mechanism of transovarian transmission is still unclear. ZIKV detected in the facet cells could imply that ZIKV also infects the nervous system organs of the mosquitoes.

Unlike *Cx*. *quinquefasciatus*, *Ae*. *aegypti* had detectable ZIKV in only the F_1_ generation, and ZIKV was not detected in the F_2_ generation in either the field strain or the laboratory strain (Table 1). ZIKV was detected in only the F_0_ generation of *Ae*. *albopictus* mosquitoes in both the field and laboratory strains. Vertical transmission of ZIKV in *Ae*. *aegypti* mosquitoes was reported by Thangamani *et al*. (2016); they found that ZIKV was transmitted to the F_1_ generation. The current study also demonstrated that ZIKV was not found in any F_1_ *Ae*. *albopictus*^52^. We therefore conclude that vertical transmission occurred in both *Cx*. *quinquefasciatus* and *Ae*. *aegypti* and not in *Ae*. *albopictus*. Further research should be conducted to explore the factors that might affect these results.

*Cx*. *quinquefasciatus* prefers to feed on animal blood, and some studies have suggested that the blood found in *Cx*. *quinquefasciatus* is 50% human, 32% bird, 12% dog, and less than 3% cat^56^. In Thailand, *Cx*. *quinquefasciatus* is found in urban and suburban areas. Feeding behavior studies in Koh Chang, Thailand showed that only 0.98% of the blood meals ingested by *Cx*. *quinquefasciatus* were human^57^. Data regarding the vertical transmission of ZIKV obtained from this study together with the feeding pattern of *Cx*. *quinquefasciatus* in Thailand indicate that *Cx*. *quinquefasciatus* mosquitoes may be a potential vector for ZIKV transmission in Thailand. Therefore, vector control strategies for addressing ZIKV outbreaks by managing mosquitoes should not focus only on *Aedes* mosquitoes; in particular, the larval control strategies should also focus on the breeding sites of *Cx*. *quinquefasciatus*. The development of vector control measures for ZIKV outbreaks in Thailand should consider both *Aedes* and *Culex* mosquitoes.

## Materials and Methods

### Ethics statement

The study was approved by the animal research ethics committee of Chulalongkorn University and adhered to the Animal Care and Use Protocol (CU-ACUP). The Faculty of Medicine, Chulalongkorn University, Bangkok, Thailand (COA No. 023/2560) approved this study, which abided by the Animals for Scientific Purposes Act and all relevant institutional policies and regulations regarding animal care and use at Chulalongkorn University. The use of hazardous agents was only initiated after approval from the institutional animal care and use committee (IACUC), Institutional Biosafety Committee (IBC), and/or Environmental Health and Safety Department. The use of human blood was approved by the Institutional Review Board of the Faculty of Medicine, Chulalongkorn University, Bangkok, Thailand (COA No. 724/2015), and the study was conducted in compliance with the international guidelines for human research protection as stated in the Declaration of Helsinki, The Belmont Report, the Council for International Organizations of Medical Sciences (CIOMS) guidelines and the International Conference on Harmonization in Good Clinical Practice (ICH-GCP).

### Mosquitoes

Laboratory and field colonies of *Ae*. *aegypti*, *Ae*. *albopictus* and *Cx*. *quinquefasciatus* mosquitoes were maintained in the Biology and Ecology Laboratory of the National Institute of Health (NIH), Department of Medical Sciences, Nonthaburi Province, Thailand, under standard conditions as follows: 28 ± 2 °C, 65–85% relative humidity, and a 12/12-hour light/dark cycle. These mosquitoes were originally obtained from eggs laid in Nonthaburi Province in Central Thailand in 2007. The populations of *Cx*. *quinquefasciatus*, *Ae*. *aegypti* and *Ae*. *albopictus* had been reared for 274, 232 and 215 generations, respectively. Field strains of *Ae*. *aegypti* and *Ae*. *albopictus* were obtained from eggs laid in Nonthaburi Province, and the F4 of those lines were used in this study. Adult mosquitoes were maintained *ad libitum* with a mixture of 5% sucrose and 5% vitamin B complex (w/v), while larvae were maintained in plastic trays and fed on minced commercial mouse food until reaching the pupal stage.

### Virus strain

The virus was provided by the National Science and Technology Development Agency of Thailand. This strain was named Zika virus/*H*. *sapiens*-tc/THA/2014/SV0127-14, and it was isolated from the blood of a patient in Thailand in 2014 and used to infect the *Toxorhynchites splendens* mosquito (1 passage). Following isolation, the virus was passed once in *Ae*. *albopictus* C6/36 cells (1 passage). This strain is from an Asian lineage. The complete genome is accession number KU681081. Viral stocks were then produced in C6/36 cells and stored at −80 °C until further use.

### Mosquito infection

Prior to artificial oral infection, the viral titer of the virus stock was calculated by fluorescent focus assay in C6/36 cells and was determined to be 1.7 × 10^6^ FFU/ml. Four- to five-day-old female *Ae*. *aegypti*, *Ae*. *albopictus and Cx*. *quinquefasciatus* mosquitoes were deprived of food for 24 hours prior to being provided with a blood meal. Starved females were fed with expired human blood from deidentified donors^58^, which was obtained from the National Blood Center, Thai Red Cross Society, Bangkok, Thailand, with ZIKV added at a concentration of 1.7 × 10^5^ FFU/ml; the mosquitoes were fed via artificial blood feeding. The female mosquitoes were allowed to feed for 45 minutes. Non-engorged females were removed, and engorged females were reared in a cage and maintained with a 5% sucrose and 5% vitamin B complex (w/v). Three days after receiving the blood meal, water containing a black plastic bow was placed into the mosquito cage for 3 days for oviposition. Mosquitoes were collected for virus detection 7 days after blood feeding (F_0_). Eggs were collected and allowed to hatch in a plastic tray. Larvae were reared to the pupal stage and then adulthood to obtain the subsequent progeny (F) and for the detection of ZIKV. Starting with the F_1_ generation, the mosquitoes were fed blood without ZIKV under the same conditions described previously.

### Viral detection in mosquitoes

There were forty pools per replicate of female (20 adults/pool) and ten pools per replicate of male (30 adults/pool) *Ae*. *aegypti*, *Ae*. *albopictus* and *Cx*. *quinquefasciatus* mosquitoes. The experiments were performed in triplicate. Each pool was ground in 1X phosphate-buffered saline (PBS) and centrifuged at 14,000 g at 4 °C for 10 minutes. The supernatant was transferred to minimum essential media (MEM) (Gibco, US) containing 2% fetal bovine serum (FBS) (Gibco, US) and maintained at −80 °C. Viral RNA was extracted from the pellets of the pooled mosquito samples, ground in a lysis solution (provided with the kit) and centrifuged; then, the supernatant was processed for RNA extraction using the Invisorb Spin Virus RNA Mini Kit (Invitec GmbH, Germany). The viral RNA samples from the mosquitoes were amplified to test for ZIKV infection by hnRT-PCR. Primers for the NS5 gene were modified from Moureau *et al*.^59^. The RT-PCR amplification reaction was set up in a final volume of 25 μl using the Superscript III One-Step RT-PCR kit, and the nested PCR was performed with 2 μl from the first reaction and 1 unit of *Taq* DNA polymerase (Fermentas, USA). *Ae*. *aegypti*, *Ae*. *albopictus* and *Cx*. *quinquefasciatus* mosquitoes that had fed on human blood without ZIKV were used as negative controls. The PCR products were analyzed via 2% agarose gel electrophoresis, stained with ethidium bromide, and visualized with Quantity One Quantification Analysis Software version 4.5.2 (Gel DocEQ System; Bio-Rad, Hercules, CA). The positive PCR products were recovered from the gel and purified using the Agarose Gel DNA Purification Kit: Invisorb Fragment CleanUp (STRATEC Molecular GmbH, Germany) following the manufacturer’s instructions. The purified DNA was sent to Macrogen, Inc. (Macrogen, South Korea) for direct DNA sequencing for confirmation of the identification of ZIKV.

### ZIKV isolation and propagation

The supernatants of the samples that tested positive by hnRT-PCR were filtered through a 0.2 µm sieve and spread in a 12-well plate with a monolayer of *Ae*. *albopictus* C6/36 cells (ATCC CRL-1660) for 1 hour. After discarding the supernatant and refreshing with 2 ml of MEM (Gibco, US) containing 10% FBS (Gibco, US), 1% penicillin (100 U/ml; Sigma-Aldrich, US), and streptomycin (100 μg/ml; Sigma-Aldrich, US) (P/S), the cells were maintained at 37 °C with 5% CO_2_. The cultures were incubated for 7 days. CPEs were checked every 24 hours for 7 days after the initial 24-hour incubation period, with observations made under an inverted microscope (Olympus, Japan).

### Mosquito salivary glands, yoke granules and eye samples

The salivary glands, midguts, yoke granules and eyes were collected at 7 days postinfection from females orally exposed to ZIKV. Anesthetized individual mosquitoes (30–40 mosquitoes per replicate) were dissected in a drop of 1X PBS on a glass slide under a stereomicroscope (Olympus, Japan). The salivary glands, embryos and head were transferred onto SuperFrost Plus microscope slides (Thermo Scientific, USA).

### ICC staining

The salivary glands, embryos and heads of infected and uninfected mosquitoes were transferred to SuperFrost Plus microscope slides (Thermo Scientific, USA), which were then air dried and fixed in 100% cold acetone before being rehydrated in graded absolute ethanol. The slides were stained with the primary rabbit-Zika virus NS1 protein antibody (GeneTex, USA) and the HRP-conjugated anti-rabbit IgG secondary antibody (Abcam, MA). The color was developed using DAB and counterstained with hematoxylin (Dako, CA), and the specimens were then examined under a light microscope (Olympus, Japan) at 100X magnification.
